# Supporting Machine Learning Model in the Treatment of Chronic Pain

**DOI:** 10.3390/biomedicines11071776

**Published:** 2023-06-21

**Authors:** Anna Visibelli, Luana Peruzzi, Paolo Poli, Antonella Scocca, Simona Carnevale, Ottavia Spiga, Annalisa Santucci

**Affiliations:** 1 Department of Biotechnology, Chemistry and Pharmacy, University of Siena, 53100 Siena, Italy; anna.visibelli2@unisi.it (A.V.); luana.peruzzi@unisi.it (L.P.); annalisa.santucci@unisi.it (A.S.); 2 POLIPAIN CLINIC, SIRCA Italian Society of Cannabis Research, 56124 Pisa, Italycarnevale.simo@gmail.com (A.S.); psicologiapolipain@gmail.com (S.C.); 3 Competence Center ARTES 4.0, 53100 Siena, Italy; 4 SienabioACTIVE—SbA, 53100 Siena, Italy

**Keywords:** machine learning, cannabis, pharmacogenetics, precision medicine, pain treatment

## Abstract

Conventional therapy options for chronic pain are still insufficient and patients most frequently request alternative medical treatments, such as medical cannabis. Although clinical evidence supports the use of cannabis for pain, very little is known about the efficacy, dosage, administration methods, or side effects of widely used and accessible cannabis products. A possible solution could be given by pharmacogenetics, with the identification of several polymorphic genes that may play a role in the pharmacodynamics and pharmacokinetics of cannabis. Based on these findings, data from patients treated with cannabis and genotyped for several candidate polymorphic genes (single-nucleotide polymorphism: SNP) were collected, integrated, and analyzed through a machine learning (ML) model to demonstrate that the reduction in pain intensity is closely related to gene polymorphisms. Starting from the patient’s data collected, the method supports the therapeutic process, avoiding ineffective results or the occurrence of side effects. Our findings suggest that ML prediction has the potential to positively influence clinical pharmacogenomics and facilitate the translation of a patient’s genomic profile into useful therapeutic knowledge.

## 1. Introduction

Chronic pain is a public health concern affecting approximately 20% of the population in North America [1], Australia [2], and Europe [3]. It is more common among women [4], elderly people [5], and the socioeconomically disadvantaged [6]. Moreover, chronic pain may have a significant impact on quality of life [7], resulting in restrictions on mobility and daily activities. Many people experience depression, anxiety and sleep disorders [8], as well as opioid dependence [9] and feelings of isolation [10]. Current therapy options have a heterogeneous approach by combining pharmacological, physical, and psychological components. However, patients do not always have a good experience with conventional treatments and most frequently mention chronic pain as their primary motivation for using and requesting alternative medical treatments, such as medical cannabis. The cannabis plant has been used for its medical and mind-altering effects for millennia. Cannabis interacts with the endocannabinoid system, a network of receptors, signaling molecules, and synthetic and degrading enzymes. The type 1 cannabinoid receptor (CB1) is mostly expressed in the central nervous system on neurons concentrated in the prefrontal cortex, hypothalamus, hippocampus, amygdala basal ganglia, and cerebellum. Cannabis’ neuropsychiatric effects are caused by heterosynaptic g-aminobutyric acid signaling [11,12]. The type 2 cannabinoid receptor (CB2) is mostly found on macrophages, B cells, and natural killer cells [13]. According to the increased knowledge of the endocannabinoid system, clinical and preclinical research efforts throughout recent decades have defined many impacts of cannabis on physiology and behavior, and a more recent study focused on its efficacy for various medical objectives [14]. Cannabis has proven to be efficient for neurological diseases, such as multiple sclerosis, Parkinson’s disease, Alzheimer’s disease, and drug-resistant epilepsy [15]; Tourette’s syndrome symptoms and spasticity [16]; rheumatoid arthritis and other rheumatic diseases [17]; fibromyalgia [18]; traditional drug-resistant glaucoma [19]; and inflammatory bowel disease [20]. Therapeutic approaches with cannabis have been attempted in the treatment of insomnia, post-traumatic stress disorder, and primary or secondary anorexia due to oncological or anti-HIV therapies. Furthermore, several studies recently showed that there is evidence to support the use of cannabinoids for the treatment of chronic or oncologic pain [21] and neuropathic pain [22]. The therapeutic effects of cannabis are a result of the presence of terpenophenolic substances known as cannabinoids. There are more than 100 cannabinoids isolated from cannabis, including cannabidiol (CBD), tetrahydrocannabinol (THC), cannabichromene (CBC), and cannabigerol (CBG) [23]. THC is the primary psychoactive constituent of cannabis, which includes feeling ‘high’, anxiety, paranoia, and cognitive deficits. THC stimulates both the CB1 and CB2 receptors, decreasing cyclic adenosine monophosphate synthesis and resulting in reduced neurotransmission [24]. Effects are observed clinically as deficits in a person’s memory, pleasure, movements, thinking, and concentration. Moreover, THC exhibits both analgesic and anti-inflammatory properties. On the contrary, CBD should not have any intoxicating or psychoactive effects, but it is important to highlight that multiple possible pharmacological targets exist for CBD, such as anticonvulsant, anxiolytic, anti-inflammatory, and neuroprotective effects [25]. Although clinical evidence supports the use of cannabis for pain, very little is known about the efficacy, dosage, administration methods, or side effects of widely used and accessible cannabis products. Additionally, there is still a lack of knowledge on the medicinal benefits of cannabinoids. A possible solution could be given by pharmacogenetics, supposing that the patient’s response to cannabinoid treatment may have a genetic background and is dependent on gene polymorphisms involved in the action, metabolism, and transport of these substances in the organism [26]. This information leads us to select potential genes whose variants may affect both the therapeutic effect and the occurrence of possible side effects and adverse reactions [27]. So far, scientists have identified numerous gene variants that determine a different cannabis therapeutic effect, affecting proteins involved in the transport, action, and metabolism of these substances. Additionally, machine learning (ML) approaches show satisfactory performance in a variety of tasks in biomedicine, including knowledge improvement in pharmacogenetics [28]. ML approaches can help aid clinical decision making by predicting treatment outcomes, guiding drug selection to avoid negative effects, and helping the identification of more effective drugs for treating differently responding subpopulations. Based on this knowledge, data from patients treated with cannabis and genotyped for several candidate polymorphic genes were analyzed using an ML method that, starting from the patient’s data collected, supports the therapeutic process, avoiding ineffective results or the occurrence of side effects.

## 2. Materials and Methods

### 2.1. Data Collection

Between November 2018 and September 2020, a cohort of 565 Caucasian patients was recruited by Azienda USL Toscana Sud-Est, San Donato Hospital (Arezzo, Italy), Department of Pain Medicine and Palliative Care [29]. The study was approved by the Tuscan Regional Ethical Committee (n. 1287) on 15 May 2018. Patients provided written consent to participate in the study and allow their genotyping after being informed of the initiative. Patients suffered from a disease defined as chronic pain for at least three months, with the presence of side effects or an inadequate response to conventional therapies. The protocols followed the Responsible Committee on Human Experimentation’s ethical requirements, as well as the 2008 revision of the Helsinki Declaration. The study’s design allowed for 4 follow-up visits every 3 months after an initial appointment, at which they were diagnosed and given a prescription for medical cannabis. At these visits, the therapy was changed according to the therapy response in the previous period. There are several medicinal cannabis varieties on the market, each characterized by a specific ratio of the amount of THC and CBD. In this study, the administered cannabis preparations were Cannabis FlosBedrocan^®^ (THC < 19%, CBD < 1%—Ministry of Health, The Netherlands), which is the most widely used cannabis, frequently used in research; Cannabis FM1^®^ (THC 13–20%, CBD < 1%—Military Pharmaceutical Chemical Institute Florence, Italy), which is one of the two varieties produced directly in Italy; Cannabis FlosBediol^®^ (THC < 6.5%, CBD < 8%—Ministry of Health, The Netherlands) and Cannabis FM2^®^ (THC 5–8%, CBD 7.5–12%—Military Pharmaceutical Chemical Institute Florence, Italy), which have a low-to-medium THC level; and FlosBedrolite^®^ (THC < 1%, CBD < 9%—Ministry of Health, The Netherlands),which is a non-psychoactive variety of cannabis. The different preparations of cannabis inflorescence were extracted under the SIFAP (Italian Association of Compound Pharmacists) method and following the Legislative Decree 9 November 2015 of the Italian Ministry of Health, which prescribes preparing cannabis extracts according to the Good Compounding Practices FU. The initial THC dosage prescribed was 5 mg per day, independent of the medicinal cannabis variety used. At the first visit, the patients were asked to sign an informed consent form, wherein they were provided information related to the therapeutic cannabis administered. Furthermore, questionnaires were offered to patients to monitor symptomatic improvement in pain and quality of life during the treatment period. One of the pain-rating scales used in epidemiologic and clinical research to measure the severity or frequency of different symptoms is the Visual Analogue Scale (VAS) [30]. The pain VAS, which ranges from “no pain” (0 value) to “worst pain” (10 value), is a unidimensional measure of pain intensity that is used to track patients’ pain progression or compare the level of pain in patients with similar diseases. A self-assessment tool called the “Hospital Anxiety and Depression Scale” (HADS) [31] was instead created to help people determine their level of anxiety and depression. There are fourteen parts to it, seven of which deal with anxiety and seven with depression.

### 2.2. Gene Analysis

Patients were genetically typed for various polymorphisms involved in drug metabolism, opioids, and pain perception. Genetic variants (single-nucleotide polymorphism: SNP) in defined genes were selected from previous publications [29] with at least one positive association with proven biological activity in individuals using cannabis for recreational purposes, given the lack of suitable indications in patients using cannabis for medical purposes. The 8 selected SNPs were as follows: MDR1/ABCB1 rs1045642; TRPV1 rs8065080; 5-UGT2B7 rs7438135; CYP3A4 rs2242480; CNR17B rs1049353; COMT rs4680; FAAH rs2295632; and CYP3A4 rs35599367. DNA was collected from a buccal swab and extracted using DNA Extract All Lysis Reagent (Applied Biosystems, Waltham, MA, USA) and SNP detection was performed by TaqMan Assay (Applied Biosystem) in an RT-PCR One-Step-Plus system (Applied Biosystem by Thermo Fisher).

### 2.3. Clinical Dataset

**Figure 1:**
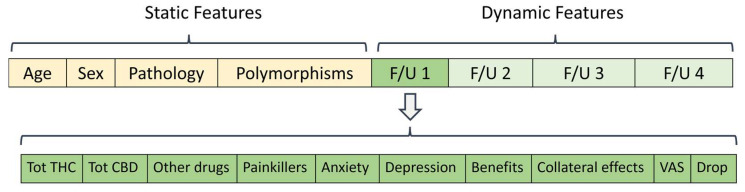
Features related to each patient, including static features in light yellow and dynamic features over the follow-up (F/U) periods in light green. In dark green, a focus on the dynamic features of each subperiod is shown.

To generate a comprehensive patient profile, clinical, genetic, and pharmacological information was collected and integrated into a multi-purpose dataset, populated by 565 subjects, uniquely identified based on an anonymous key. Each patient in the dataset was defined by two different types of features: the ones that changed over the treatment period (dynamic) and the ones that remained constant (static). Static features include age (from 2 to 94 years old); sex (male/female); pathology (CNS pathologies, rheumatoid arthritis, inflammatory diseases, neuropathic pain, others); and patient polymorphisms. Dynamic features include, for each treatment subperiod, total daily CBD and THC dose expressed in milligrams; painkillers assumption (yes/no); other drugs taken (yes/no); VAS (from 0 to 10); HADS results, benefits (yes/no); and collateral effects (yes/no). A column named “Drop” was also added, which includes patients who decided to drop out of the treatment early for various reasons, such as a lack of pain reduction or the occurrence of side effects (Figure 1).

### 2.4. Machine Learning Method

Regression analysis was performed in this study, which examined the relationship between the dependent variables (target), which belong to a continuous domain, and the independent variables in a dataset. The job of the modeling algorithm is to find the best mapping function from input variables to the continuous output variable. EXtreme Gradient Boosting (XGBoost 1.7.6) [32] was selected as the best model, a machine learning technique that produces a predictive learner in the form of a set of weak predictive models, allowing the optimization of an arbitrary differentiable cost function. The method employs the gradient-descent algorithm to minimize errors in sequential models. Decision trees were selected as weak predictors, which tend to outperform all other algorithms in manipulating small and structured data. Regularization parameters can be added and tuned with XGBoost and are highly effective in reducing computing time providing optimal use of memory resources. To evaluate and report the performance of the XGBoost model, the mean absolute error (MAE) was calculated. The MAE is a metric commonly used because the units of the error score correspond to the units of the predicted target value. The MAE does not give different weight to different types of errors and the scores increase linearly as the error increases. It is determined by averaging the absolute error value, which is the difference between an expected and predicted value. Standard deviation (SD) was also calculated, a statistic that measures the dispersion of a dataset relative to its mean. The bigger the deviation within the data collection, the further the data points deviate from the mean; thus, the higher the standard deviation, the more spread out the data.

## 3. Results

The use of computational tools has gained increasing importance, thanks to their ability to process biological data at an unprecedented speed, revealing information and patterns hidden within. Machine learning models represent a crucial component in the development of algorithms and come with great opportunities to reduce cost, increase reproducibility, and save time. Because of that, in this study, we implemented an XGBoost prediction of the optimal combination of THC and CBD doses to be prescribed to each patient, based on the genetic, pharmacological, and clinical data information described above.

### 3.1. Data Pre-Processing

**Figure 2:**
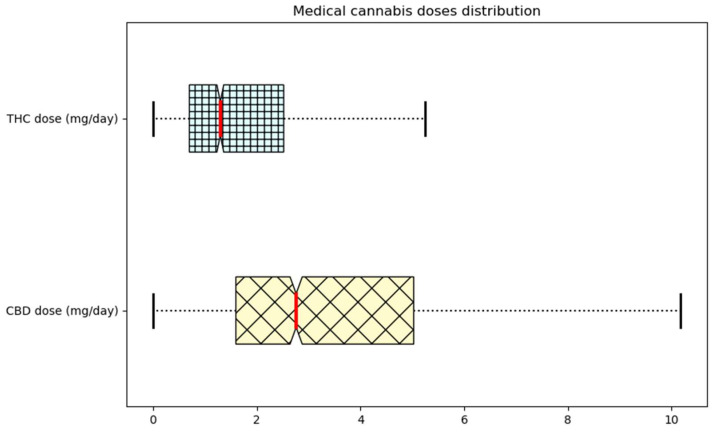
Medical cannabis dose distribution range. Boxplot of CBD and THC mg/day distribution. Lower and upper box boundaries represent the 25th and 75th percentiles, respectively; the red line inside the box shows the median; and the lower and upper error lines highlight the 10th and 90th percentiles, respectively. Outliers’ data falling outside the 10th and 90th percentiles were not reported.

ML algorithms are sensitive to the range and distribution of attribute values. Data extreme values can mislead the training process, resulting in less accurate models and poorer results. To remove these possible outliers, we first represented (Figure 2) and analyzed the distribution of THC and DBC doses/day in the dataset. All the subjects were under treatment with medical cannabis, a combination of CBD and THC from 0.01 to 15 and 20 mg, respectively, with a mean of 2.7 mg for THC and 1.6 mg for CBD. A dose greater than 10 mg was rarely prescribed. Therefore, we did not consider THC daily dose prescriptions over 9 mg or CBD doses over 8 mg.

**Figure 3:**
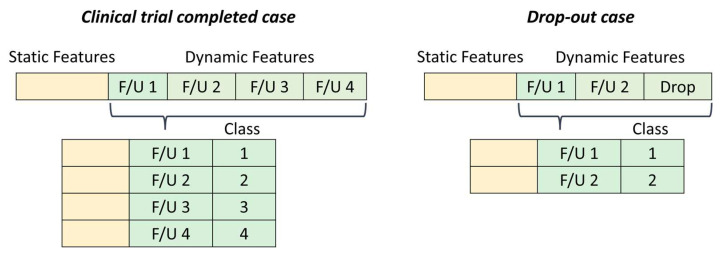
Patients who decided to attend all the follow-up visits were completely described by four rows. Drop-out case examples include a patient who dropped the treatment after the second follow-up visit, represented therefore by only two rows.

Additionally, the “Drop” column, which includes patients who decided to drop out of the study, caused the dataset to contain many empty cells. To solve this problem, we decided to split the dataset by considering each patient as many times as the number of follow-up periods, as shown in Figure 3.

Moreover, to maintain the temporal aspect and avoid the problem of identical rows with different cannabinoid doses, a “Class” column was added to distinguish each follow-up period, incrementing the dataset to 1823 rows. The dataset was then pre-processed to make it suitable for further computational analysis. Each categorical value was encoded with values between 0 and n classes-1, while numerical columns were scaled to the [0, 1] range.

### 3.2. XGBoost Prediction

**Figure 4:**
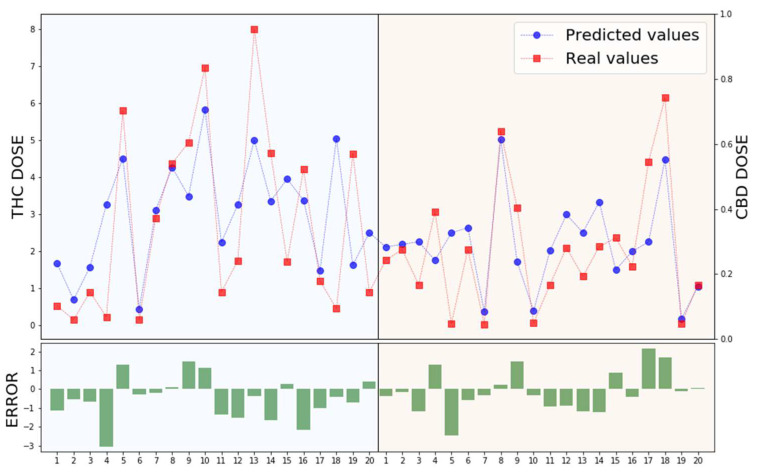
Real observations are shown as single red points within the plot, while predicted values are represented as single blue points. THC and CBD mg/day predicted vs. real values are plotted on the left and the right side of the plot, respectively. In the *X*-axis, 20 values for each cannabinoid are reported. The *Y*-axis represents daily dose values. The lower graph includes error bars for each prediction, which represents the variation of the corresponding coordinate of the point.

Pre-processed data were then divided into the training set and the test set. The training set consisted of 80% of the dataset and was used to make the model learn the hidden features. The remaining 20% belonged to the test set to test the model after completing the training. The XGBoost hyperparameters were selected after a grid, which consisted of trying every possible configuration to find the parameter set that guarantees the highest accuracy. We implemented a model with 1300 trees with a maximum depth of 12, while we used the default values for the minimum sum of instance weight needed in a child. A feature selection step was not necessary as the model was already able to prioritize the most important features and filter out irrelevant ones. The network was trained and tested on 20 runs, each using a different dataset split. Moreover, the MAE and SD were calculated to evaluate the performance of the model, resulting in a MAE value equal to 1.01 mg with a SD of 0.04 mg. Figure 4 reported the distance between a subset of predicted and real values both for THC and CBD doses, including error bars of each prediction.

**Figure 5:**
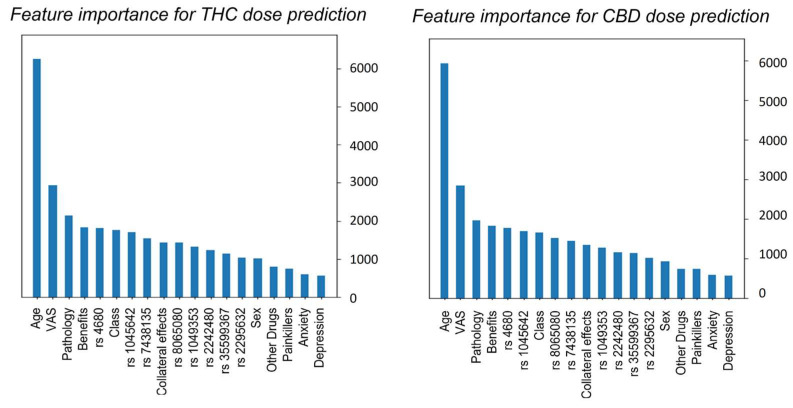
Feature importance bar graph for THC and CBD daily dose prediction on the left and on the right, respectively. All the features are listed and sorted by their importance. The *X*-axis displays feature names used as the input of the ML model, while the *Y*-axis show which features attribute the most predictive power to the model.

From the graphs in Figure 4 emerges an extraordinary ability of the model to accurately predict most of the values, while still showing an increase in error for the prediction of the extreme values caused by the unbalanced data distribution. Because our aim remains the possibility to understand the importance given by the genetic features in the prediction, a feature importance bar graph is also reported (see Figure 5).

## 4. Discussion

In this study, we developed a powerful XGBoost algorithm to predict the appropriate cannabis therapeutic dose. In addition, we showed those that are the main predictors in our model through bar graphs of feature importance. Despite the difficulties in properly quantifying the effects of cannabis treatment and the intricacy of cannabis pharmacology, both bar graphs highlight the importance of age as the first factor in determining the correct dose of cannabinoids. Indeed, the influence of age on the effect of cannabis on the brain has been extensively studied, although the results do not offer a conclusive answer on the type of role age has. The VAS parameter shows high importance for delineating the right treatment. This outcome was very encouraging and in line with previous studies that state that medical cannabis could be a viable alternative to traditional treatments for chronic pain [33,34]. On the contrary, no gender differences are observed in the effects of cannabinoids on chronic pain. Research on gender differences is a growing field of interest as cannabinoid-based therapies are being evaluated for various pain disorders. However, no definitive conclusions can be made about sex differences regarding efficacy or the mechanisms mediating these possible differences [35]. Surely, there is a need for more studies investigating gender variations in the regulation of endocannabinoid signaling to create medications that target the endocannabinoid system [36]. Furthermore, high impact in the prediction is given to the pathology suffered. In this study, patients were grouped into five different pathology classes: central nervous system disorders, inflammatory rheumatic diseases, migraine headaches, spinal disorders, and other pathologies. Although there is strong evidence that cannabinoids are effective in treating rheumatic disease and central nervous system disorders, the clinical evidence remains lacking and has not progressed significantly over the last few years. Clinical trials that provide positive endpoints and evidence that medicinal cannabis should be considered a frontline therapeutic remain largely elusive [37,38]. Similarly, the long-term effects of cannabis in treating spinal disorders are poorly understood. Other active cannabinoids besides THC are potential therapeutic agents for treating symptoms of spinal disorders [39], yet evidence regarding effectiveness is limited due to low-quality studies with small sample sizes, leaving a sizable knowledge gap in this area. Differently, according to recent studies, the endocannabinoid system is involved in migraine mitigation through several central and peripheral pathways [40]. Cannabinoids have a specific prophylactic effect in migraines thanks to their ability to inhibit platelet serotonin release and their peripheral vasoconstrictor effect [41].

Additionally, it is possible to evaluate the impact of specific genetic variants in patients and explain part of the great inter-individual variability observed in pain reduction. Part of this is significantly correlated with four polymorphic candidate genes which have been studied to be involved in the therapeutic activity of cannabis [26]: ABCB1 rs1045642, TRPV1 rs8065080, UGTB7 rs7438135, and COMT rs4680.

The rs1045642 polymorphism in the ABCB1 gene (ATP-binding cassette subfamily B member 1, OMIM: 171050) has been linked to changes in drug response and disease susceptibility. This SNP has been extensively studied in relation to cannabis dependence, but the findings from these investigations have been contradictory [42,43]. It was suggested that the ABCB1 rs1045642 polymorphism might influence THC psychoactive effects and an individual’s vulnerability to dependence [44]. Recently, a study also revealed a significant association with steroid-resistant nephrotic syndrome [45]. Another relevant genetic variant is the rs8065080 polymorphism of the TRPV1 gene (transient receptor potential cation channel subfamily V member 1, OMIM: 602076). This SNP has been associated with an increased risk of hypertension [46] and is believed to influence an individual’s perception of salt at levels above the threshold [47]. Moreover, the TRPV1 rs8065080 polymorphism has been found to significantly affect heat pain thresholds in patients with neuropathic pain and they have also been linked to changes in mechanical pain sensitivity and mechanical hypesthesia [48]. The rs7438135 variant of the UGT2B7 gene (UDP glucuronosyltransferase family 2 member B7, OMIM: 600068) has been proven to play a role in opioid withdrawal symptoms [49]. Additionally, this SNP is involved in the glucuronidation process of morphine. The rs4680 polymorphism of the COMT gene (catechol-O-methyltransferase, OMIM: 116790) has been extensively studied in relation to THC’s impact on memory, attention, and in the reduction in decision-making abilities [50]. The COMT rs4680 variant appears to modulate the association between cannabis use and psychotic disorders, particularly in individuals who were exposed to cannabis at an early age [51]. Furthermore, COMT rs4680 has been suggested to increase the risk of cannabis-use disorders [52].

On the contrary, the four other gene variants analyzed (CYP3A4 rs2242480, CYP3A4 rs35599367, CNR17B rs1049353, and FAAH rs2295632) as other variables (other drugs and painkillers taken, depression, and anxiety conditions) remained very far from being significant. The rs2242480 variant of the CYP3A4 gene (cytochrome P450 family 3 subfamily A Member 4, OMIM: 124010) has been suggested to be associated with an increased risk of drug addiction among the Chinese Han population [53]. Additionally, this SNP has been found to elevate the risk of severe withdrawal symptoms in patients undergoing methadone maintenance treatment [54]. Another genetic variant is the rs35599367 polymorphism of the CYP3A4 gene, which has been shown to reduce CYP3A4 mRNA expression in the liver [55]; it has also been associated with plasma simvastatin concentrations [56]. The rs1049353 polymorphism in the CNR1 gene (cannabinoid receptor type 1, OMIM: 114610) has been extensively studied, but the previous literature has provided conflicting results regarding its association with cannabis use and subjective effects. While some studies emphasize a strong link with cannabis-dependence symptoms [57], others have not found a significant association between CNR1 rs1049353 and substance abuse or cannabis dependence [58]. Moreover, this polymorphism has been implicated in the abuse of other substances, such as alcohol-use disorder and heroin dependence [59]. The rs2295632 variant in the AAH gene (fatty acid amide hydrolase gene, OMIM: 602935) leads to the inhibition of the resulting enzyme, resulting in reduced degradation of endocannabinoids [60]. Recent studies have also indicated that this SNP can influence susceptibility to certain multifactorial disorders [61]. Furthermore, the homozygous T/T genotype of FAAH rs2295632 has been statistically associated with type 2 diabetes mellitus [62].

This initial study is limited by its small sample size, heterogeneous variables, which can introduce confounding bias, and lack of specific quantified risks associated with the different genetic variants. As a result, there is a lack of evidence on the potential adverse effects of the chronic medical use of cannabis and a lack of focus on patients who dropped out of treatment with cannabis mainly because of the lack of pain reduction and side effects. Moreover, cannabis products are commercially available with multiple routes of administration with different durations of effects and relative safety. These characteristics need to be considered as the pharmacokinetics and side effects are strongly dependent on both routes of administration and compound formulation [63]. In this study, we instead decided to exclude this information and included the daily dose in mg regardless of the route of administration. Therefore, accurate research must assess functional outcomes in addition to reduced pain scores and evaluate long-term tolerability and alternate routes of administration. Other limitations of the study include a lack of placebo control, which makes it harder to be certain that the outcome was caused by the experimental treatment and not by other variables. Future studies will aim to address these limitations.

## 5. Conclusions

Pain relief or the occurrence of side effects from taking cannabis is difficult to estimate, and the clinical management of cannabis treatments is left to the experience of clinicians who need to tailor therapy individually according to the patient’s general condition. In this study, we demonstrated the ability of ML methods to evaluate the role of specific genetic polymorphisms in patients suggesting that genetic inheritance is a significant descriptive factor of response variability to cannabis. The model was based on data collected in a heterogeneous, standardized database developed and implemented to collect information on patients treated with medical cannabis. This approach will possibly contribute to clarifying the pharmacokinetics and pharmacodynamics of cannabis, increasing the clinical community’s trust in its therapeutic application, and supporting their choices with a tool that can provide the importance of each component. Our findings suggest that ML prediction has the potential to positively influence clinical pharmacogenomics and facilitate the translation of a patient’s genomic profile into useful therapeutic knowledge. We are aware that predicting a more individualized treatment with the help of the knowledge of patients’ genetic traits will only be achievable by substantially extending the polymorphic gene panel and the number of their polymorphisms. Many challenges remain open, requiring the development of alternative strategies to complement/improve existing techniques.

## Data Availability

The data presented in this study are available on request from the corresponding author. The data are not publicly available because we are evaluating an eligible publicly accessible repository.
